# Proteomic and Transcriptomic Analyses Indicate Reduced Biofilm-Forming Abilities in Cefiderocol-Resistant *Klebsiella pneumoniae*

**DOI:** 10.3389/fmicb.2021.778190

**Published:** 2022-01-03

**Authors:** Jinfeng Bao, Lu Xie, Yating Ma, Ran An, Bing Gu, Chengbin Wang

**Affiliations:** ^1^Department of Clinical Laboratory, The First Medical Centre, The PLA General Hospital, Beijing, China; ^2^Laboratory Medicine, Guangdong Provincial People’s Hospital, Guangdong Academy of Medical Sciences, Guangzhou, China; ^3^College of Medical Technology, Xuzhou Medical University, Xuzhou, China

**Keywords:** *Klebsiella pneumoniae*, cefiderocol, resistance, biofilm, siderophore

## Abstract

The advent of cefiderocol provides hope for the clinical treatment of multi-drug resistant gram-negative bacteria (GNB), especially those with carbapenem resistance. Resistance of *Klebsiella pneumoniae* to cefiderocol can be enhanced by acclimatization. In the present study, we collected cefiderocol resistant *K. pneumoniae* isolates during a 36-day acclimatization procedure while increasing the cefiderocol concentration in the culture medium. Strains were studied for changes in their biological characteristics using proteomics and transcriptomics. A decrease in biofilm formation ability was the main change observed among the induced isolates. Downregulation of genes involved in biofilm formation including *hdeB, stpA, yhjQ, fba, bcsZ, uvrY, bcsE, bcsC*, and *ibpB* were the main factors that reduced the biofilm formation ability. Moreover, downregulation of siderophore transporter proteins including the iron uptake system component *efeO*, the tonB-dependent receptor *fecA*, and ferric iron ABC transporter *fbpA* may be among the determining factors leading to cefiderocol resistance and promoting the reduction of biofilm formation ability of *K. pneumoniae*. This is the first study to investigate cefiderocol resistance based on comprehensive proteomic and transcriptomic analyses.

## Introduction

Multidrug-resistant *Klebsiella pneumoniae* (MDR-KP) became an important cause of high morbidity and mortality in severely infected patients in healthcare settings during recent years (Lee et al., 2016). Extended spectrum beta-lactamases (ESBLs) and carbapenemases are now recognized as main resistance factors in MDR-KP because they can hydrolyze almost all cephalosporin and carbapenem antibiotics (Daoud et al., 2017; Wright et al., 2017). Even more seriously, the linear rise in incidence of carbapenem-resistant *K. pneumonia* (CR-KP) generates a serious antibiotic management challenge associated with the increasingly widespread occurrence of carbapenem-resistance genes, such as those encoding *K. pneumoniae* carbapenase (*bla*_KPC_), New Delhi metallo-β-lactamase (*bla*_NDM_), and oxacillinase enzymes (*bla*_OXA–48_) (Sheng et al., 2013; Jean et al., 2015; Lee et al., 2016). New antibiotics or alternatives to antibiotic treatment are urgently needed in order to address this global clinical problem.

Cefiderocol is a novel ferrocephalosporin with siderophore capacities and the first iron-carrying cephalosporin to be evaluated in Phase III clinical trials (Falagas et al., 2017; Monogue et al., 2017). It can penetrate the bacterial outer membrane upon binding of ferric iron and utilization of bacterial iron transporters (Kong et al., 2019; Negash et al., 2019). This “Trojan horse” strategy allows for cefiderocol to reach an elevated concentration in the periplasm of bacterial cells, to then bind to and inhibit penicillin-binding proteins (PBPs), prevent cell wall synthesis and ultimately cause death. Cefiderocol affects cellular osmotic resistance and exerts high-level antimicrobial activity against carbapenem resistant gram-negative bacteria (GNB), such as *Escherichia coli*, *Acinetobacter baumannii* and *Pseudomonas aeruginosa* (Ghazi et al., 2017; Nguyen et al., 2018; Isler et al., 2020; Johnston et al., 2020; Simeon et al., 2020; Bassetti et al., 2021). Cefiderocol is considered a promising drug for the clinical treatment of infections by carbapenem-resistant bacteria and as such will save patients’ lives.

However, cefiderocol belongs to the class of beta-lactam antibiotics and works by interfering with the structural cross-linking of peptidoglycans in bacterial cell walls. The production of beta-lactamases, changes in drug targets, changes in pore proteins that reduce intra-cellular drug accumulation, or the activity of efflux pumps are four possible factors contributing to bacterial resistance to beta-lactam antibiotics (Blair et al., 2015; Meletis, 2016). Biofilm formation is known to be closely associated with drug resistance as well, since it adequately protects bacteria against external stressors including changes in pH and osmotic pressure or nutrient deficiencies (Fux et al., 2005; Lewis, 2007; McCarty et al., 2012). The bacteria first lose activity, become fixed, and then colonize from the surface to grow, thus preventing drugs from entering the bacteria in the process of biofilm formation (Stewart and Costerton, 2001; Sharma et al., 2019). Although some studies have demonstrated that cefiderocol can inhibit biofilm formation by MDR-GNB, the molecular mechanism of reduced biofilm formation by *K. pneumoniae* and the role of cefiderocol or cefiderocol resistance has not been elucidated (Harrison and Buckling, 2009; Pybus et al., 2020). This need to be investigated to better preserve cefiderocol efficacy and design methods for prevention of resistance development.

Proteomics and transcriptomics are powerful methods to study the adaptation mechanisms of bacteria to various antibiotics (Sianglum et al., 2011; Vranakis et al., 2012; Lata et al., 2015; Khan et al., 2017; Sun et al., 2017; Sharma et al., 2018). We applied proteomics and transcriptomics to investigate the biological characteristics and molecular mechanisms of cefiderocol resistance in *K. pneumoniae* which evolved during a laboratory-based cefiderocol resistance induction method. This method allowed precise culture condition and time control and excluded the risk of genetic transformation. We collected cefiderocol resistant *K. pneumoniae* isolates during a 36-day acclimatization while increasing the cefiderocol concentration over time and performed of proteomic and transcriptomic analyses. To the best of our knowledge, this is the first study to provide proteome and transcriptome evidence about the adaptation mechanisms of *K. pneumoniae* to cefiderocol, hoping that the results could help explore novel therapeutics targets against cefiderocol resistance.

## Materials and Methods

### Bacterial Strains and Cefiderocol Treatment

Wild type (WT) strain *K. pneumoniae* NTUH-K2044 donated by Taiwan University (Taiwan, China) was used in this study. The bacterial suspensions were adjusted to 0.05 McFarland (MF) with saline solution, then 1 μL bacterial fluid and 500 μL lysogeny broth (LB) medium were added to each well of a 48-well plate containing different concentrations of cefiderocol (MCE, United States). The plate was incubated at 37 °C, shaking at 180 rpm for 24 h, and the cefiderocol concentration where bacterial suspension was at an OD_600_ below 0.1 was recorded as the Minimum inhibitory concentrations (MICs) using the methodology described in Andrews (2001). The MIC of WT strain was experimentally determined as 0.0625 μg/mL. Three single colonies of WT strains were cultured overnight and then diluted 1:100 in fresh LB medium with or without sub-MIC doses (0.03 μg/mL) of cefiderocol for 24 h incubation at 37°C with agitation, the MIC was determined again. This same procedure was repeated until the MIC of cefiderocol added up to 20 μg/mL (36 days), being 320-fold that of the WT strains (0.0625 μg/mL). Thereafter, three colonies of the final resistance level of the cefiderocol-treated strains and three WT strains were selected for the subsequent proteome and transcriptome analyses, respectively.

### Growth Curve Analysis and *in vitro* Competition Experiment

To compare the basic growth rate of the WT and cefiderocol-treated strains, two kinds of strains were inoculated on blood agar plates and cultured at 37°C for 18 h. Monoclonal colonies were selected, and the concentration of the bacterial suspension was adjusted to 10^6^ CFU/mL. One microliter of the bacterial suspension was added into a well of a 96-well plate containing 200 μL LB broth and cultured at 180 rpm at 37°C. The A_600_ value of the bacterial solution was recorded every hour, and the evolution of the A_600_ value was recorded for 18 h. The experiment was repeated 10 times in both groups.

The WT and cefiderocol-treated strains in logarithmic growth phase were collected to compare their *in vitro* competition ability, with the concentration of each strain diluted to 1.5 × 10^3^ CFU/mL in a 1:1 ratio. After mixing the resistant bacteria with susceptible bacteria, they were added to 10 mL LB liquid medium and cultured at 37°C and 180 rpm for 18 h. The total amount of the WT and cefiderocol-treated strains was counted by 10-fold gradient dilution method. One hundred microliter of bacterial suspension was plated on LB agar and a separate LB agar plate containing 10 g/L cefiderocol. Both plates were cultured at 37°C for 18 h. Colonies were counted on the plates to determine the competitive ability of the resistant strains.

### Transmission Electron Microscopy Analysis

To observe the morphological changes at the cellular level of cefiderocol-treated bacteria vs. WT bacteria, transmission electron microscopy (TEM) was carried out. Pre-processing steps before TEM were as follows. A bacterial pellet was washed with phosphate buffer solution (PBS, pH 7.4) and fixed with 2.5% glutaraldehyde. After PBS rinsing, cells were incubated in 1% OsO_4_ at 25°C for 2 h, and then rinsed with PBS. The sample was then dehydrated with a series of graded ethanol: water solutions (30, 50, 70, 80, 90, 95, and 100%) and finally converted to pure acetone. The dehydrated samples were embedded in Embed 812 and incubated in a 65°C oven for 48 h. Finally, specimens were cut into ultrathin sections (60–80 nm) with a microplate analyzer, stained with lead uranyl acetate citrate for 15 min, and observed by HT7700 TEM (Hitachi, Japan).

### Biofilm Formation Ability Test

The crystal violet (CV) staining method was used to examine the biofilm formation ability with regard to biomass accumulation according to the method modified from Sun et al. (2019). The WT and cefiderocol-treated strains were inoculated on blood agar plates and cultured at 37°C for 24 h. Monoclonal colonies were selected and the concentration of the bacterial suspension was adjusted to 1.5 × 10^7^ CFU/mL. A total of 200 μL bacterial liquid was added into the 96-well plate and cultured at 37°C for 4, 8, 12, 16, and 24 h. Then, 200 μL of crystal violet solution was added into the well and culture was continued for 20 min. The suspension was washed 3 times with sterile normal saline and dried at room temperature. The absorbance of the sample well at 590 nm was measured with a microplate analyzer. Prior to measuring absorbance of retained crystal violet, 200 μL of glacial acetic acid was added to each well. Each strain was repeatedly tested using 10 wells.

### Carbon Metabolism

Carbon source utilization of *K. pneumoniae* was determined using the Biolog-ECO technique described by Wen et al. (2021). Metabolism of 31 carbon sources was determined using the method described by Yang et al. (2019). Each WT or cefiderocol-treated strain was incubated in LB broth at 37°C and 220 rpm for 12 h, and washed with the same volume of PBS for 3 times. Then cell suspensions were diluted to 0.5 McF with NS. Total of 150 μL bacterial diluent was added to the well of the ECO enzymatic plate (Biolog, United States) and then cultured at 25°C in darkness. OD_590_ and OD_750_ were measured every 24 h and recorded continuously for 6 days. The OD_590–750_ value of each well indicates the metabolic intensity of the strain to each carbon source, while average well color development (AWCD) represents the average metabolic capacity to total carbon sources. The AWCD of each well represents Σ (OD_590–750_)/31 (31 carbon sources in the ECO plate). The single carbon source metabolism ratio equals (single carbon source OD_590–750_) × 100%/Σ (OD_590–750_ of each carbon source). The total carbon sources included six major carbon sources: esters, amines, acids, carbohydrates, alcohols, and amino acids. Three replicates were used for each strain.

### Proteomic Analysis

4D label-free quantitative proteomics and a combined LC-MS/MS analysis were performed for proteomic analysis of *K. pneumoniae* from both WT and cefiderocol-treated strains according to the methods described by Ma et al. (2020) and Sun et al. (2020). The Maxquant search engine (v.1.5.2.8) was used to process the resulting MS/MS data (Cox and Mann, 2008). Based on the genomic data of *K. pneumoniae* NTUH-K2044, tandem mass spectrometry was performed from the UniProt database. Enzyme digestion method was set as Trypsin (Full) and the number of missing cleavages was set as 2. The minimum length of the peptide segment was 6 amino acid residues. The maximum number of peptide modifications was 3. The mass error tolerance of the primary parent ion was 10 PPM, and that of the secondary fragment ion was 0.02 Da. Carbamidomethyl (C) was specified to fixed modification, Oxidation (M), Acetyl (N-terminus), Met-loss (M), Met-loss + Acetyl (M) were set to variable modification. Protein, peptide, and the false discovery rate (FDR) identified by peptide-Spectrum Match (PSM) were all adjusted to 1%. To obtain high quality results, database search results need further data filtering. The accuracy of the FDR in the three levels of spectrum, peptide and protein identification was specified to 1%. To identify a protein, at least one unique peptide was required. Peptides sizes are ranging between 7 and 20 amino acids in accordance with the general rules of enzymatic hydrolysis and higher-energy C-trap dissociation (HCD) fragmentation based on trypsin. When the *P*-value were less than 0.05, statistical significance was recognized, proteins with a fold change ≥ 1.5 or ≤ 0.67 were considered differentially expressed proteins (DEPs). Then, Gene Ontology (GO) classification and Kyoto Encyclopedia of Genes and Genomes (KEGG) pathway enrichment and corresponding clustering analysis were performed for the differential expressed proteins.

### Transcriptome Analysis

Total RNA of the WT and cefiderocol-treated strains was extracted for transcriptomic analysis. The RNA-seq sequencing and assembly were carried out according to the method described by Dai et al. (2019). RNA was firstly extracted from the bacteria, then followed by strict quality control of the RNA samples and accurate detection of RNA integrity and total RNA concentration mainly using Agilent 2100 Bioanalyzer (Agilent Technologies, CA, United States). After the extracted RNA is qualified, ribosomal RNA (rRNA) in total RNA is removed to obtain mRNA. Subsequently, the mRNA fragments were randomly interrupted into short fragments by fragmentation buffer, and the library was built in a chain-specific way (Parkhomchuk et al., 2009). After qualified library inspection, Illumina NovaSeq 6000 sequencing is performed after pooling different libraries according to the requirements of effective concentration and target offline data amount to obtain the sequence information of the fragment.

Based on the genome of *K. pneumoniae* NTUH-K2044, the sequence was screened and mapped by Bowtie2 (v.2.3.4.3), the mismatch was set to 2 and default parameters were used for the rest of the software (Langmead and Salzberg, 2012). Gene expression levels were defined by HTSeq (v.0.9.1), then calculated according to the acquired reads using Fragments Per Kilobase per Million mapped reads (FPKM) values (Garber et al., 2011). The differentially expressed genes (DEGs) were analyzed by DESeq2 (v.1.20.0) for the two groups (Anders and Huber, 2012). DEGs were identified based on the combined criteria of | log2 (fold change) | > 1, *P* ≤ 0.05 and FDR ≤ 0.01 after normalization of the expression abundance. Then, GO classification and KEGG pathway enrichment and corresponding cluster analysis were performed for the DEGs using the corresponding database as reference. Please refer to Supplementary File 1 for more details.

### Statistical Analysis

SPSS 25.0 was used for analysis of growth curve, *in vitro* competition ability, biofilm formation ability and carbon metabolism, with *P* < 0.001 as the threshold of significance. Data were expressed as the mean ± standard deviation. Significant differences between the WT group and cefiderocol-treated group were determined based upon Two independent sample *t*-test.

## Results

### *Klebsiella pneumonia* Acquires Resistance to Cefiderocol Through a Multistage Process

**FIGURE 1:**
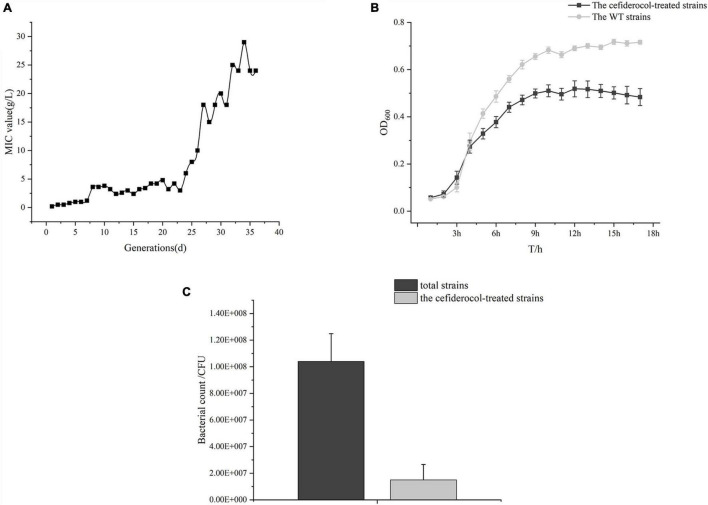
Change of induced cefiderocol concentration *in vitro* for 36 days and general changes after cefiderocol induction. **(A)** Change of induced cefiderocol concentration in 36 consecutive days. **(B)** Growth curve of the WT strains and the cefiderocol-treated strains. **(C)** Comparison of *in vitro* competitive ability between the WT and cefiderocol-treated strains.

Firstly, we compared changes in cefiderocol-treated concentration and MIC. The overall MIC value of *K. pneumonia* NTUH-K2044 to cefiderocol increased 320-fold from 0.0625to 20 μg/mL after 36-day induction in our experiment. The induction concentration of cefiderocol was at a low level (under 5 μg/mL) 20 days before induction, and then increased almost linearly from 21 to 30 days (Figure 1A), suggesting that the evolution of cefiderocol resistance of *K. pneumonia* followed a pattern of rapid increasing after breaking through the bottleneck.

### General Biological Phenotypic Changes

**FIGURE 2:**
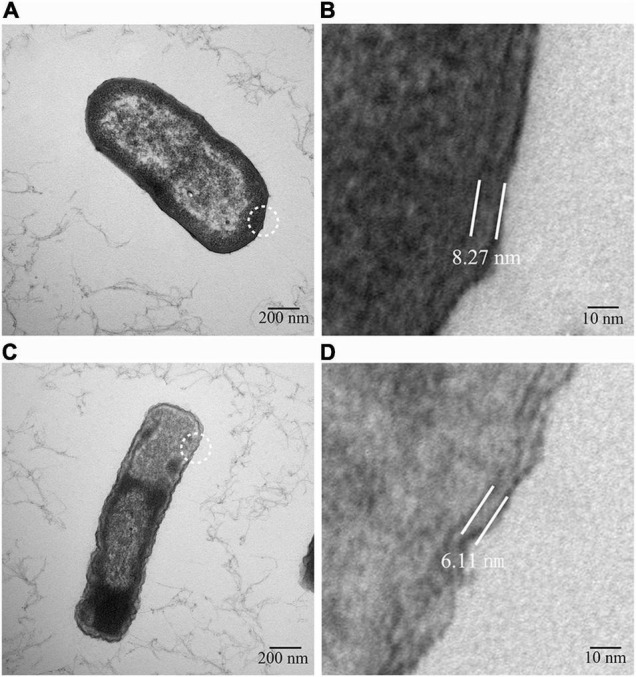
Transmission electron micrograph. **(A)** Strain morphology of the WT strain. **(B)** Cell wall thickness of the WT strain. **(C)** Strain morphology of the cefiderocol-treated strain. **(D)** Cell wall thickness of the WT strain of the cefiderocol-treated strain. **(B,D)** Are the enlarged images in the white circles of **(A,C)**, respectively. The cell wall thickness of the WT strain was about 8.72 nm, while that of the cefiderocol-treated strain was about 6.11 nm.

The basic biological changes of the strains before and after cefiderocol induction were compared, including the size and morphology of the strain and *in vitro* competitiveness. The cefiderocol-treated strains exhibited a significantly slower growth rate (Figure 1B and Supplementary Table 1) as compared to the WT strain. At the same time, the cefiderocol-treated strains were more stretched than WT strains (Figure 2 and Supplementary Figure 1). Interestingly, the cell walls of cefiderocol-treated strains became thinner (Figures 2B,D) and their *in vitro* competition ability was decreased compared to WT strains (Figure 1C). All the changes observed above indicated that the acquisition of resistance goes at the expense of fitness.

### Reduction in Capacity of Biofilm Formation

**FIGURE 3:**
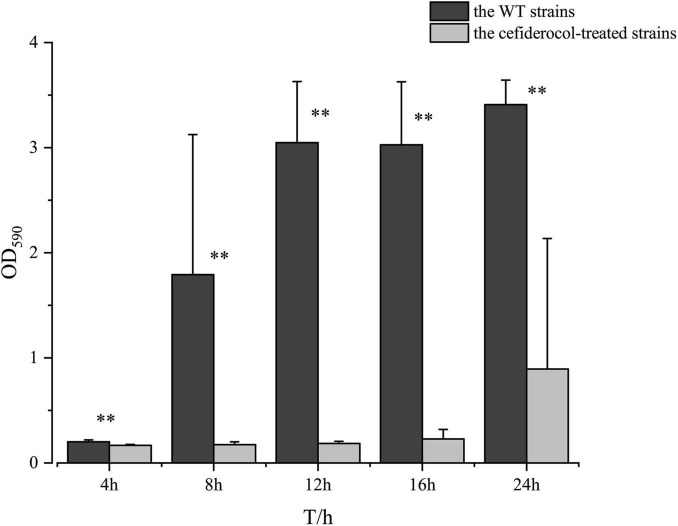
Comparison of biofilm formation ability between the WT and cefiderocol-treated strains over time (4, 8, 12, 16, and 24 h). The absorbance value at OD_590_ was used to compare the biofilm forming ability of the WT and cefiderocol-treated strains with two independent sample *t*-test (^**^P < 0.001). Error bars represent standard deviations from the means.

Interestingly, it was found that the ability of the biofilm formation ability of the cefiderocol-treated strain was significantly lower than that of the WT strains at each time period, suggesting that cefiderocol could effectively inhibit the biofilm-forming ability of *K. pneumonia*, even if it acquires resistance to cefiderocol. In addition, the biofilm formation ability of strains from the two groups both increased gradually with time (Figure 3).

### The Regulation of Carbon Metabolism

**FIGURE 4:**
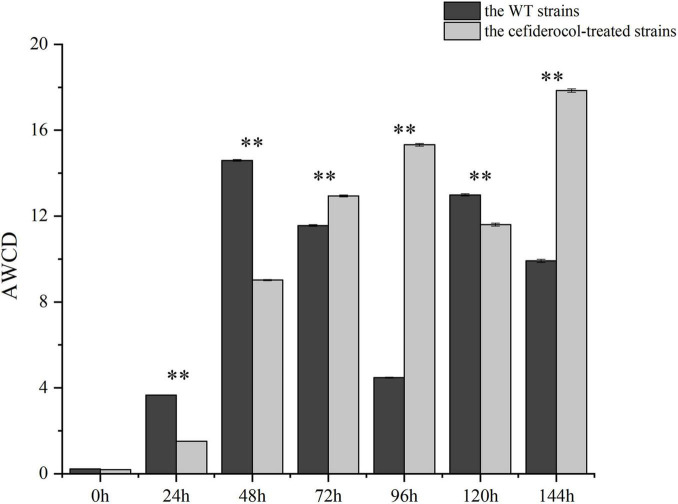
Comparison of total carbon metabolism ability between the WT and cefiderocol-treated strains in 6 time periods (24, 48, 72, 96, 120, and 144 h). The average well color development (AWCD) was used as an indicator of the total carbon utilization capacity of microorganisms with two independent sample *t*-test (^**^P < 0.001). Error bars represent standard deviations from the means.

The quantification of carbon metabolites can be used to characterize the degree of microbial demand for external nutrients. The average well color development (AWCD) was used as an indicator of the total carbon utilization capacity of the strains under study. As shown in Figure 4 and Supplementary Table 2, AWCD of cefiderocol-treated strains was significantly lower than that of the WT strain during 0–48 h (*P* < 0.001), indicating that the WT strain grew more rapidly with increasing nutrient levels. However, The AWCD of the WT strains decreased during 72–96 h, while AWCD of the cefiderocol-treated strains increased during that same period. AWCD was similar between the WT and cefiderocol-treated strains at 120 h, and AWCD of the cefiderocol-treated strain began increasing markedly, suggesting that the cefiderocol-treated strains might enhance carbon metabolism gradually.

**FIGURE 5:**
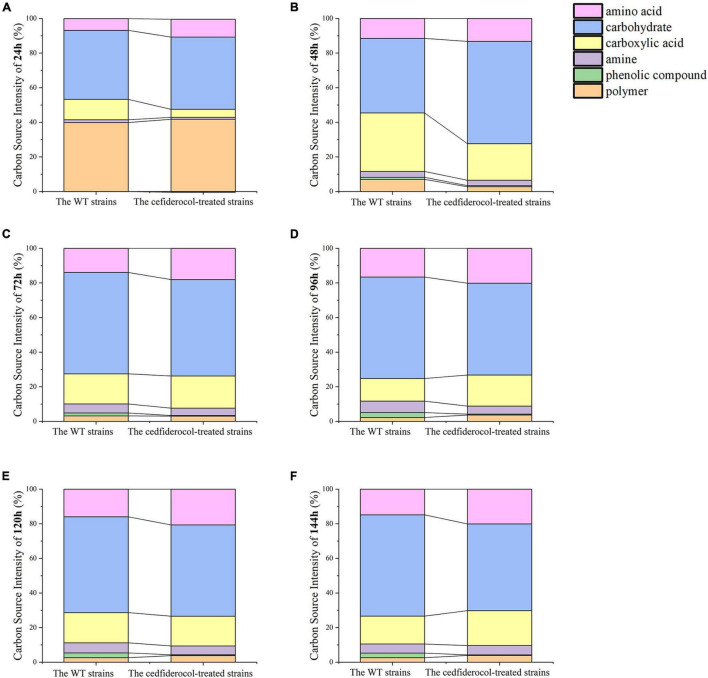
**(A–F)** Utilization rates of 6 kinds of carbon sources for the WT strains and cefiderocol-treated strains in 6 time periods (24, 48, 72, 96, 120, and 144 h), respectively.

In addition, the utilization of the WT and cefiderocol-treated strains to six carbon sources (carbohydrates, amino acids, carboxylic acids, amines, phenolic compounds and polymers) was estimated by Biolog-ECO experiments. As shown in Figures 5A–F and Supplementary Table 3, the WT and cefiderocol-treated strains have different utilization of carbon sources. The WT strains mainly uses carbohydrates, carboxylic acids and amino acids from high to low, while the cefiderocol-treated strains refer to carbohydrates, amino acids and carboxylic acids, respectively. Moreover, the utilization rate of carbohydrate and amino acid of the cefiderocol-treated strains was higher than that of the WT strains.

### Transcriptomic and Proteomic Data for Cefiderocol-Treated *Klebsiella pneumonia*

**FIGURE 6:**
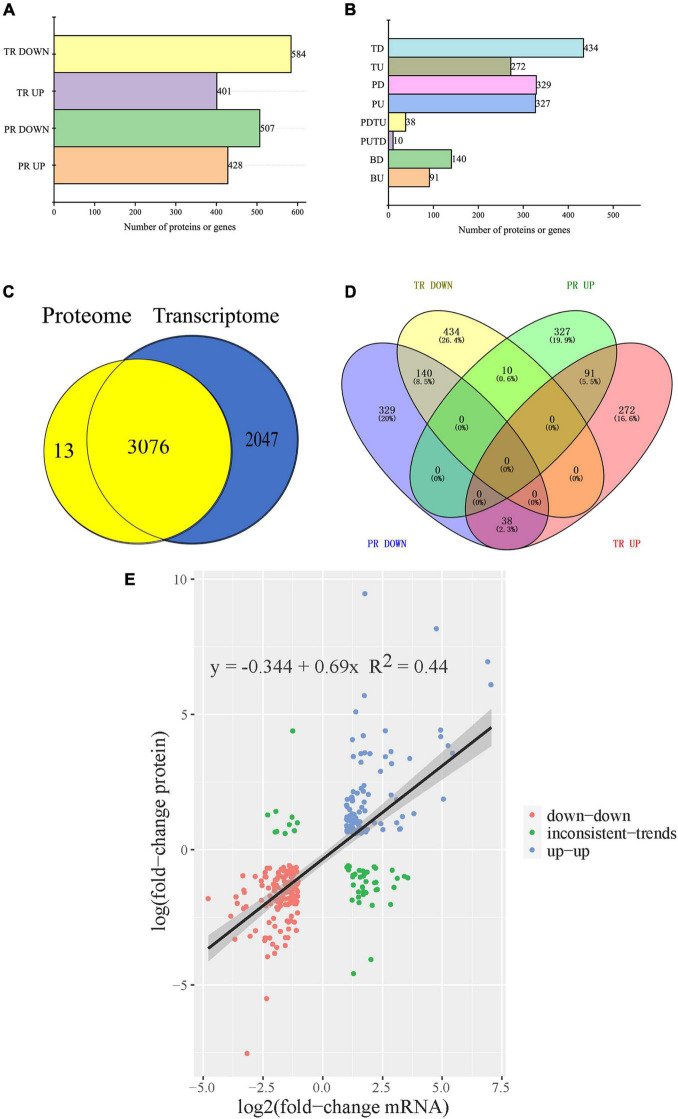
Integration analysis of proteome and transcriptome. **(A)** The profile of differentially transcribed genes (DEGs) and differentially expressed proteins (DEPs) of cefiderocol-treated strains compared with the WT strains. **(B)** The classification of DEGs and DEPs. **(C)** Comparison of the number of identified transcripts and proteins. **(D)** Venn diagram of the number of DEPs and DEGs in proteomics and transcriptome. **(E)** Scatter plot of genes and its corresponding proteins expression in cefiderocol-treated strains vs. the WT strains. BU, transcript level and protein level both up-regulated. PUTD, transcript level down-regulated and protein level up-regulated. TU, transcript level up-regulated and protein level not regulated. PU, transcript level normal and protein level up-regulated. PDTU, transcript level up-regulated and protein level down-regulated. BD, transcript level and protein level both down-regulated. TD, transcript level down-regulated and protein level not regulated. PD, transcript level normal and protein level down-regulated. PR, proteomics. TR, transcriptomics.

Proteomic and transcriptomic analyses were performed to define the internal mechanism of physiological differences and resistance of cefiderocol-treated strains. The proteomic responses of cefiderocol-treated *K. pneumonia* were analyzed by 4D label-free and a combined LC-MS/MS quantitative proteomic approach. A total of 3,089 proteins was identified and 2,775 proteins were quantified. Among these, 935 DEPs (428 up-regulated and 507 down-regulated) were observed in cefiderocol-treated strains after data filtering (fold change ≥ 1.5 or ≤ 0.067, *P* ≤ 0.05, FDR ≤ 0.01) when compared to the WT strain (Figure 6B). The characteristics of cefiderocol resistant *K. pneumonia* at the level of the transcriptome was studied using RNA-seq. The relative gene expression levels were evaluated by comparing with the WT strain and a total of 5,123 genes were quantified. After data filtering (| log2 (fold change) | ≥ 1and *P* ≤ 0.05, FDR ≤ 0.01), a total of 985 DEGs (401 up-regulated and 584 down-regulated) were screened out in cefiderocol-treated strains when compared to the WT strain (Figure 6B).

**FIGURE 7:**
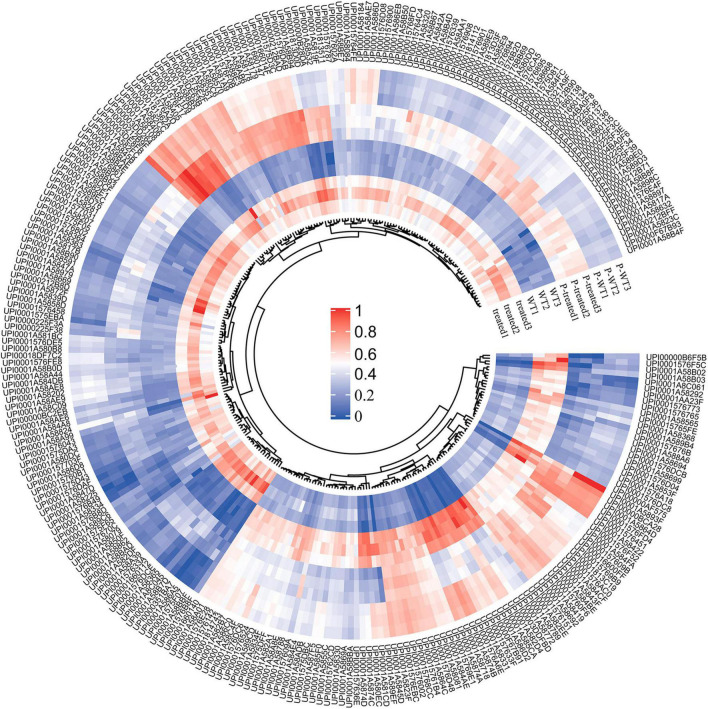
Heatmap of the relative abundances of the DEGs shared by proteome and transcriptome. WT1–3 and treated1–3 represent the six strains in transcriptome, while P-WT1–3 and P-treated1–3 are the six strains in the proteome. The gradient from blue to red indicates the relative degree expression of these DEGs. The outer circle is the ID of these DEGs.

**FIGURE 8:**
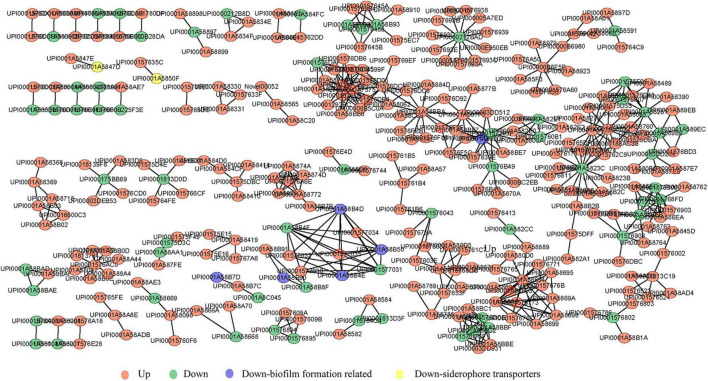
Interaction networks for up and down-regulated proteins (genes) of cefiderocol-treated strains at both the protein and transcription levels. Circular nodes in red and green indicate up and down-regulated protein mapped in cefiderocol-treated strains, respectively. Biofilm formation related proteins and siderophore transporters proteins which we focused on are marked in blue and yellow, respectively.

By comparing the 3,089 proteins and 5,123 genes that seemed differentially expressed, 3,067 proteins simultaneously differing identified in their transcriptome and proteome were traced (Figure 6A) and 279 of these were differentially expressed (Figure 6C). 140 up-regulated and 91 down-regulated genes were found among the 279 common DEGs, and 10 genes were up-regulated in proteome but down-regulated in transcriptome (Figure 6D). Moreover, 38 genes were down-regulated in proteome but up-regulated in transcriptome. As shown in Figure 6E, correlation coefficient of the expressions of protein and gene were 0.44, indicating that the expression of proteins and genes were not always consistent, which is involved with many complicated factors (Roichman et al., 2021). For example, mRNA translation is also regulated by a number of factors, such as microRNA, or the precursors of the translated protein need to be modified and activated. The relative expression abundance of DEGs which were up-regulated and down-regulated in both proteome and transcriptome in each sample is shown in Figure 7 and Supplementary Figure 2. The interactions of these co-up and down regulated proteins are shown in Figure 8, which was visualized through Cytoscape (v.3.6.1, Shannon et al., 2003).

GO functional enrichment analysis of proteome and transcriptome were plotted (Supplementary Figure 3). In terms of biological process, DEGs and DEPs were mainly involved in metabolism and molecular synthesis of various compounds, such as carbohydrates and aromatic compounds. In terms of cellular composition, DEGs and DEPs were mainly associated with membrane synthesis, cell part and intracellular part. In terms of molecular function, DEGs and DEPs mostly exhibited the function of binding, oxidoreductase activity, transferase activity and hydrolase activity.

KEGG analysis showed that the DEGs and DEPs were mainly involved in microbial metabolism in diverse environments, biosynthesis of secondary metabolites, ABC transporter expression, fructose and mannose metabolism, biosynthesis of amino acids, two-component system expression, quorum sensing, alanine, aspartate and glutamate metabolism, lysine degradation, amino sugar and nucleotide sugar metabolism, biosynthesis of cofactors, and phosphotransferase system (PTS) expression (Supplementary Figure 4).

**FIGURE 9:**
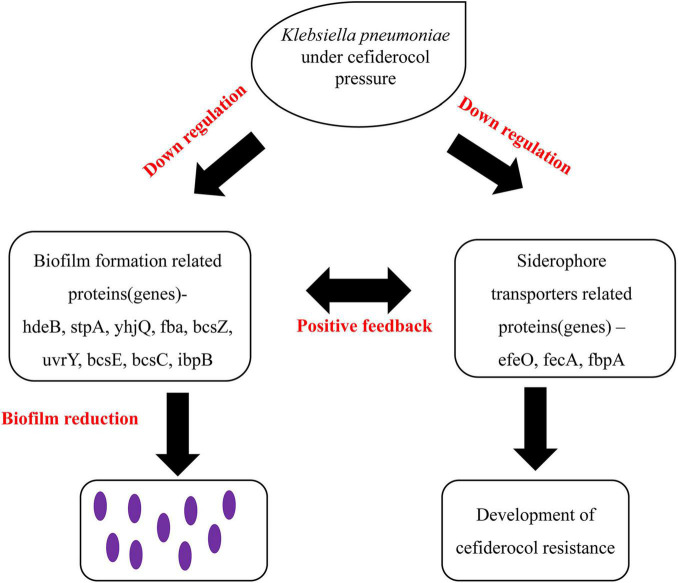
Proposed model based on the positive feedback effect between the reduction of biofilm formation ability and siderophore transporters proteins.

In the present study, biofilm formation ability was reduced significantly in cefiderocol-resistant *K. pneumonia*, which attracted our attention on biofilm formation-related proteins (genes). After critical analysis of the DEGs and DEPs in both proteome and transcriptome, we found 12 down regulated proteins (genes) that belonged to different categories but all played a critical function in the regulation of biofilm formation (Table 1 and Figure 9). These proteins (genes) are acid stress chaperone *hdeB*, DNA-binding protein *stpA*, cellulose synthase operon protein *yhjQ*, fba protein *fba*, cellulase *bcsZ*, barA-associated response regulator *uvrY*, cellulose biosynthesis protein *bcsE*, cellulose synthase operon protein *bcsC*, small heat shock protein *ibpB*, iron uptake system component *efeO*, tonB-dependent receptor *fecA*, and ferric iron ABC transporter *fbpA*.

**TABLE 1:** Main differently expressed biofilm formation-related proteins (genes) in proteomics and transcriptomics.

Gene ID	Gene name	Protein name	Proteome		Transcriptome	
			Fold change	*P*-value	Log2 (fold change)	*P*-value
UPI0001A58B7D	hdeB	Acid stress chaperone HdeB	0.0054	4.215E−05	–3.157	3.100E−07
UPI0001A58862	stpA	DNA-binding protein	0.0817	9.217E−05	–1.9137	2.876E−10
UPI0001A58B90	yhjQ	Cellulose synthase operon protein YhjQ	0.1040	0.0010	–2.4015	1.110E−28
UPI0001A582DF	fba	Fba protein	0.1405	0.0001	–1.9916	4.154E−11
UPI0001A58B4E	bcsZ	Cellulase	0.251	1.041E−05	–2.1727	6.271E−31
UPI0001A58668	uvrY	BarA-associated response regulator UvrY (GacA, SirA)	0.3043	0.0001	–1.0396	5.008E−32
UPI0001A58B50	bcsE	Cellulose biosynthesis protein BcsE	0.3503	4.397E−05	–2.0212	6.603E−18
UPI0001A58B4D	bcsC	Cellulose synthase operon protein C	0.4968	0.0022	–2.3667	4.676E−33
UPI0001A58B67	ibpB	Small heat shock protein IbpB	0.6239	0.0022	–2.2122	0.0174
UPI0001A5850F	efeO	Iron uptake system component EfeO	0.105	0.0002	–1.45254	2.125E−06
UPI0001A5898C	fecA	TonB-dependent receptor	0.1982	0.0054	–1.0432	5.238E−06
UPI0001A5847D	fbpA	Ferric iron ABC transporter, iron-binding protein	0.4167	0.0005	–1.0935	0.0016

## Discussion

Currently, cefiderocol resistance in clinical practice is mainly due to the production of β-lactamase enzymes, and the combination of *PER* and *NDM* is important factors leading to cefiderocol resistance (Ito et al., 2019). The activity of cefiderocol against MDR GNB is clinically interesting since this antibiotic rapidly penetrates cells via their iron channels. Also, cefiderocol is very stable and withstands serine- and metal-carbapenemases (Yamano, 2019). Klein et al. (2021) found heterogeneous mutations in the *cirA* gene in carbapenemase-producing *Enterobacter cloacae*, which encoded a catecholate siderophore receptor, contributing to resistance to cefiderocol. However, the exact mechanism(s) of cefiderocol resistance remain(s) unclear.

Here, we successfully obtained cefiderocol resistant *K. pneumonia* strains, which showed a 320-fold increase in MIC compared with the WT strains. Certain biological changes occurred in cefiderocol resistant *K. pneumoniae*, mainly a reduction in their growth rate, changes in thallus morphology and a reduced ability to form biofilms. Previous studies (Dobias et al., 2017; Aoki et al., 2018; Hackel et al., 2018; Ito et al., 2018; Yamano, 2019) have demonstrated that cefiderocol has a strong ability to inhibit the growth of both susceptible and resistant bacteria. The cell shape of bacteria is largely determined by the structure of the cell walls that surround them, and a multi-protein machine known as the rod system has long been associated with rod shape determination in bacteria (Rohs et al., 2021). It was found in our study that cell shape-determining proteins *MreC* and *MreB*, which belong to the Rod system, were up-regulated at the protein level, making the shape of cefiderocol resistant *K. pneumoniae* longer and thinner (Wachi et al., 1989). The decrease in cell wall thickness of cefiderocol-resistant strains mainly related to the induction of drug efflux pumps or changes in biofilm formation relative to empty carrier control (Chuang et al., 2015). Conventional β-lactam resistance proteins (genes) including efflux pump membrane transporter *acrD*, *OmpA* family proteins and multidrug resistance proteins *mdtA*, *mdtB*, *mdtC*, and *mdtD* were significantly up-regulated in both protein level and transcription level, which might play important roles in the cefiderocol resistance of *K. pneumoniae*.

In addition, our results demonstrated that cefiderocol resistance of *K. pneumoniae* is potentially associated with changes in carbon metabolism, which is important in nutrition and for the growth and reproduction of microorganisms. Lopatkin et al. (2021) also found that alterations in central carbon and energy metabolism led to lower basal respiration levels, which prevents the induction of the tricarboxylic acid cycle mediated by antibiotics, thus avoiding metabolic toxicity. Moreover, the WT and cefiderocol resistant strains both preferred carbohydrates for ATP production, which is the main and direct energy source for vital movements (Taillefer and Sparling, 2016; Verschueren et al., 2019). Efficient and rapid use of carbohydrates promotes the growth and survival of bacteria.

### The Internal Mechanism of the Decrease of Biofilm Forming Ability

Biofilms are aggregates of single or multiple bacterial species encased in a protective extracellular matrix, which is typically composed of secreted exopolysaccharides (EPS), environmental DNA (eDNA), proteins, surfactants, lipids and water (Stewart, 2002; Artini et al., 2013; Tan et al., 2015). The process of biofilm formation includes surface attachment and movement, formation of microcolonies, maturation and eventual diffusion (Parrino et al., 2019; Singh et al., 2019). This ability is widely distributed among microbes and contributes to a broad spectrum of defense mechanisms (Hall-Stoodley et al., 2004; Piperaki et al., 2017). We found that the biofilm formation ability of cefiderocol-treated *K. pneumoniae* was significantly lower than that of the WT strains.

A group of proteins and genes involved in the formation of biofilm were found to be down-regulated in cefiderocol resistant *K. pneumoniae.* These proteins (genes) are acid stress chaperone *hdeB*, DNA-binding protein *stpA*, cellulose synthase operon protein *yhjQ*, fba protein *fba*, cellulase *bcsZ*, barA-associated response regulator *uvrY*, cellulose biosynthesis protein *bcsE*, cellulose synthase operon protein *bcsC*, small heat shock protein *ibpB*, iron uptake system component *efeO*, tonB-dependent receptor *fecA*, and ferric iron ABC transporter *fbpA*.

Acid stress chaperone *hdeB* and small heat shock protein *ibpB* influence biofilm formation of GNB through stress response and surface hydrophobicity (Zhang et al., 2007). Nucleoid-associated protein *stpA* represses biofilm formation through its interaction with the H-NS K57N (Hong et al., 2010). Mutant strains deficient in H-NS or *stpA* generated biofilms poorly comparing with the WT isogenic strain (Belik et al., 2009). Moreover, the over-expression of genes *fba* and *uvrY* could promote bacterial biofilm formation, which has been reported in related analyses (Müsken et al., 2010; Mitra et al., 2013; Liu et al., 2017). In addition, biofilm regulator BssR was found up-regulated in protein level and transcription level, which inhibited the formation of bacterial biofilms by influencing cell signaling (Domka et al., 2006).

The down regulation of bacterial cellulose synthesis (Bcs) proteins *bcsC*, *bcsE*, *bcsZ* and cellulose synthase operon protein *yhjQ* was confirmed both by proteomics and transcriptomics, which all need cCyclic-di-GMP to act as second messengers to regulate biofilm formation (Le Quéré and Ghigo, 2009). cCyclic-di-GMP is known as the key molecule that regulates bacterial biofilm formation (Hengge, 2009). cCyclic-di-GMP is considered to be an intracellular signaling molecule that coordinates the transition of sessile to a motile lifestyle or vice versa (Römling et al., 2013). The correlation between high concentrations of cCyclic-di-GMP in cells and bacterial biofilms has been demonstrated for many bacteria (Simm et al., 2004). cCyclic-di-GMP regulates a variety of factors in the process of biofilm formation, including biofilm dispersion, surface adhesion, flagella rotation, type IV pili contraction, exopolysaccharide production, secondary metabolite production, antimicrobial resistance and other stress responses (Römling et al., 2013).

Considering that cefiderocol is a siderophore cephalosporin and biofilm-forming microorganisms may utilize bacterial siderophores to acquire iron (Harrison and Buckling, 2009; Wilson et al., 2016). Siderophore antibiotics are an attractive strategy to overcome the low permeability of the outer membrane of GNR with the combination of antimicrobial molecules. In the “Trojan horse” approach of cefiderocol treatment, TonB-dependent receptors (TBDRs) are involved in not only drug conjugate transport but also uptake of essential nutrients such as iron. It is reported that *PiuA* and *PirA* and their homologs in the TBDRs of *A. baumannii* and *P. aeruginosa* are involved in the uptake of siderophore-beta-lactam drug conjugates (Luscher et al., 2018). Several proteins and genes related to acquisition of iron ions, iron uptake systems and ferric aerobactin receptors have been reported to promote biofilm formation (Kang and Kirienko, 2018; Li et al., 2021). It was found in our study that iron uptake system component *efeO*, tonB-dependent receptor *fecA*, and ferric iron ABC transporter *fbpA* were down-regulated in both proteomic or transcriptomic analyses. This may be one of the important factors of cefiderocol resistance, but independent confirmation is required. Hence, although the resistant strains acquired resistance to cefiderocol, the biofilm formation ability of which significantly decreased, suggesting the viability and development space of resistance was greatly reduced. This may be one of the reasons why iron-carrier antibiotics become a focus of research in recent years.

Based on the above findings, we hypothesize that there existing a positive feedback effect between the reduction of biofilm formation and siderophore transporter proteins. This is based on the theory that the acquisition of iron is a necessary condition for the formation of biofilms and the formation of biofilms promotes the production of siderophores (Chua et al., 2014; Kang and Kirienko, 2018; Figure 9). The down regulation of iron carrier proteins in cefiderocol resistant strains may have led to the reduction in iron acquisition and further the reduction of biofilm formation ability. The reduction of biofilm-forming ability in turn promoted the down-regulation of iron carrier proteins, this kind of positive feedback may be one of the important factors leading to the reduction of biofilm formation ability of cefiderocol resistant strains.

## Conclusion

We used proteomics and transcriptomics to investigate the molecular features of selected cefiderocol-resistance of *K. pneumoniae* and found that changes of *K. pneumoniae* effected by cefiderocol acclimatization were mainly reflected in the ability of biofilm formation, which was consistent with the down-regulation of biofilm formation proteins (genes), including acid stress chaperone *hdeB*, DNA-binding protein *stpA*, cellulose synthase operon protein *yhjQ*, fba protein *fba*, cellulase *bcsZ*, barA-associated response regulator *uvrY*, cellulose biosynthesis protein *bcsE*, cellulose synthase operon protein *bcsC*, small heat shock protein *ibpB*, iron uptake system component *efeO*, tonB-dependent receptor *fecA*, and ferric iron ABC transporter *fbpA*. The resistance of *K. pneumoniae* to cefiderocol was significantly related to the down-regulation of siderophore transporters proteins and we speculate that there existing a negative feedback effect between the reduction of biofilm formation ability and siderophore transporters proteins, which may be a crucial factor for the reduction of biofilm formation ability of *K. pneumoniae* when it simultaneously acquired cefiderocol resistance. We will continue to explore this aspect in the future research. We hope these findings could provide valuable information for further analysis of resistance mechanisms of *K. pneumoniae* to cefiderocol and may be helpful to the mitigation and control of antibiotic resistance.

## Data Availability Statement

The datasets presented in this study can be found in online repositories. The names of the repository/repositories and accession number(s) can be found below: NCBI—PRJNA764778; iProX—IPX0003528000.

## Author Contributions

BG and CW designed and managed the project. JB performed all the experiments. LX and YM did the analysis of proteomics and transcriptome analysis. JB and RA wrote the manuscript. All authors reviewed the manuscript.

## Conflict of Interest

The authors declare that the research was conducted in the absence of any commercial or financial relationships that could be construed as a potential conflict of interest.

## Publisher’s Note

All claims expressed in this article are solely those of the authors and do not necessarily represent those of their affiliated organizations, or those of the publisher, the editors and the reviewers. Any product that may be evaluated in this article, or claim that may be made by its manufacturer, is not guaranteed or endorsed by the publisher.

## Supplementary Material

Supplementary Figure 1 Changes of bacterial growth state. **(A,C)** Show the observation of colony under oil microscope (×100) with gram stain; **(B,D)** show the morphological differences of strains under electron microscope without ultrathin sections.

Supplementary Figure 2 Heatmap of the relative abundances of the DEGs shared by proteome and transcriptome. **(A)** Heatmap of the relative abundances of the 140 down-regulated DEGs. **(B)** Heatmap of the relative abundances of the 91 up-regulated DEGs. The red and green columns represent genes that are up-regulated and down-regulated in the proteome or transcriptome, respectively. The gradient from blue to red indicates the relative degree expression of these DEGs.

Supplementary Figure 3 GO analysis of the DEGs shared by proteome and transcriptome. The green, blue and red fonts on the left represent biological function, molecular composition and cellular function, respectively. Red and blue columns represent the number of down-regulated and up-regulated DEGS, respectively.

Supplementary Figure 4 KEGG analysis of the DEGs shared by proteome and transcriptome. Red and blue columns represent the number of down-regulated and up-regulated DEGS, respectively.
