# Supplementary figures and images for: Proteomic and Transcriptomic Analyses Indicate Reduced Biofilm-Forming Abilities in Cefiderocol-Resistant Klebsiella pneumoniae
Source: Front Microbiol. 2022 Jan 3;12:778190. doi: 10.3389/fmicb.2021.778190 (PMC8762213; doi:10.3389/fmicb.2021.778190)

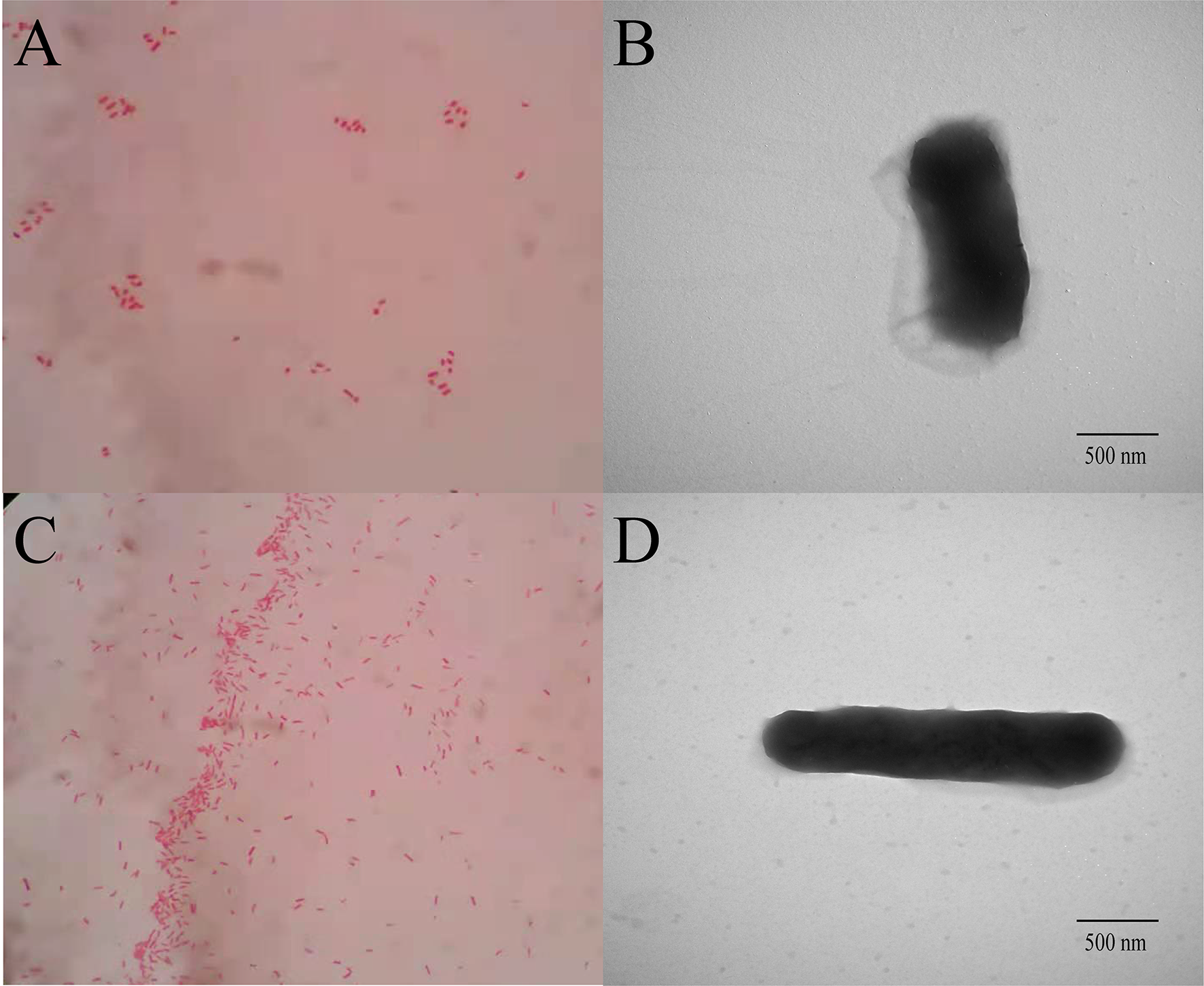

Supplement: Supplementary Figure 1 — Changes of bacterial growth state. (A,C) Show the observation of colony under oil microscope (×100) with gram stain; (B,D) show the morphological differences of strains under electron microscope without ultrathin sections. [file Image_1.TIF]

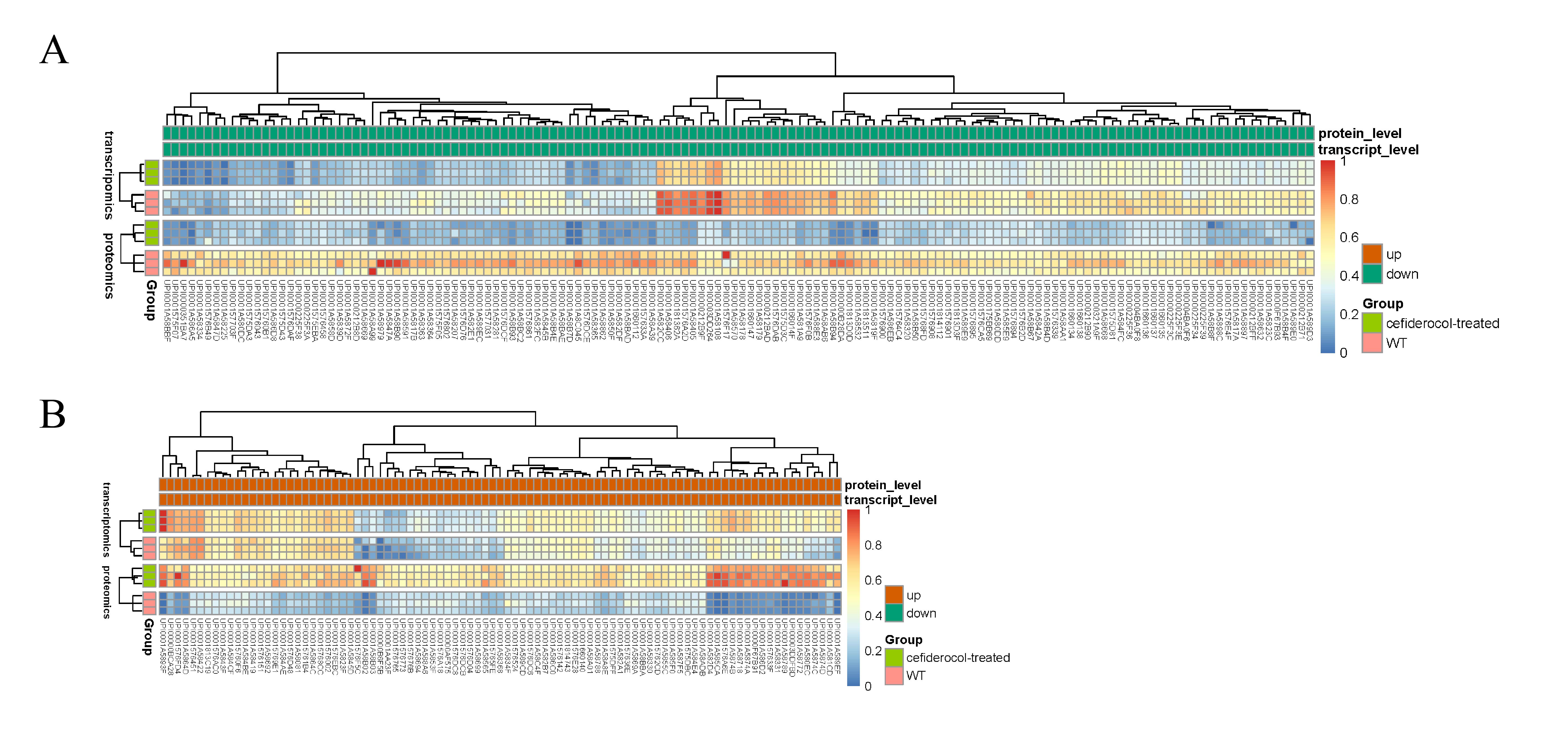

Supplement: Supplementary Figure 2 — Heatmap of the relative abundances of the DEGs shared by proteome and transcriptome. (A) Heatmap of the relative abundances of the 140 down-regulated DEGs. (B) Heatmap of the relative abundances of the 91 up-regulated DEGs. The red and green columns represent genes that are up-regulated and down-regulated in the proteome or transcriptome, respectively. The gradient from blue to red indicates the relative degree expression of these DEGs. [file Image_2.JPEG]

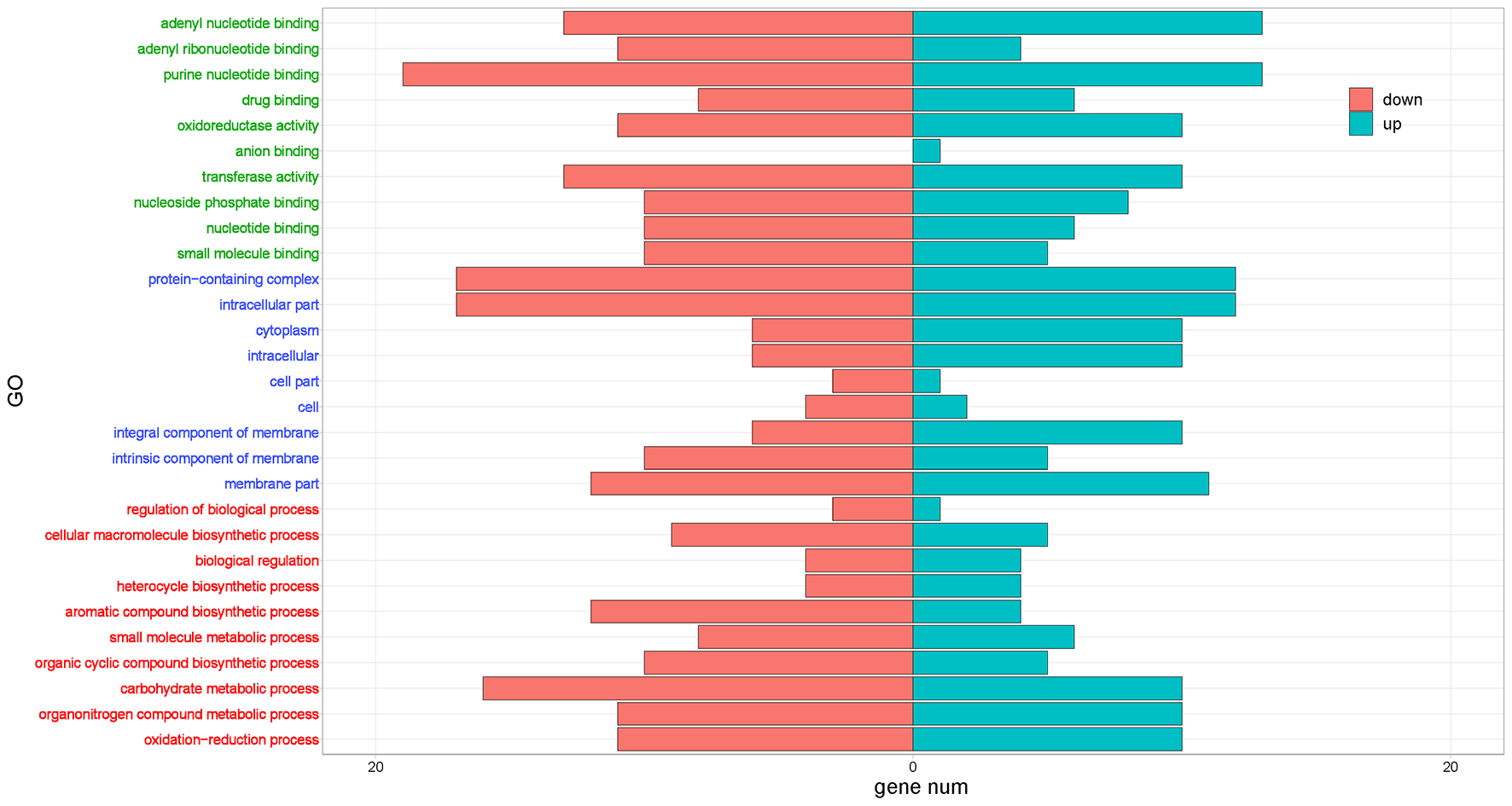

Supplement: Supplementary Figure 3 — GO analysis of the DEGs shared by proteome and transcriptome. The green, blue and red fonts on the left represent biological function, molecular composition and cellular function, respectively. Red and blue columns represent the number of down-regulated and up-regulated DEGS, respectively. [file Image_3.TIF]

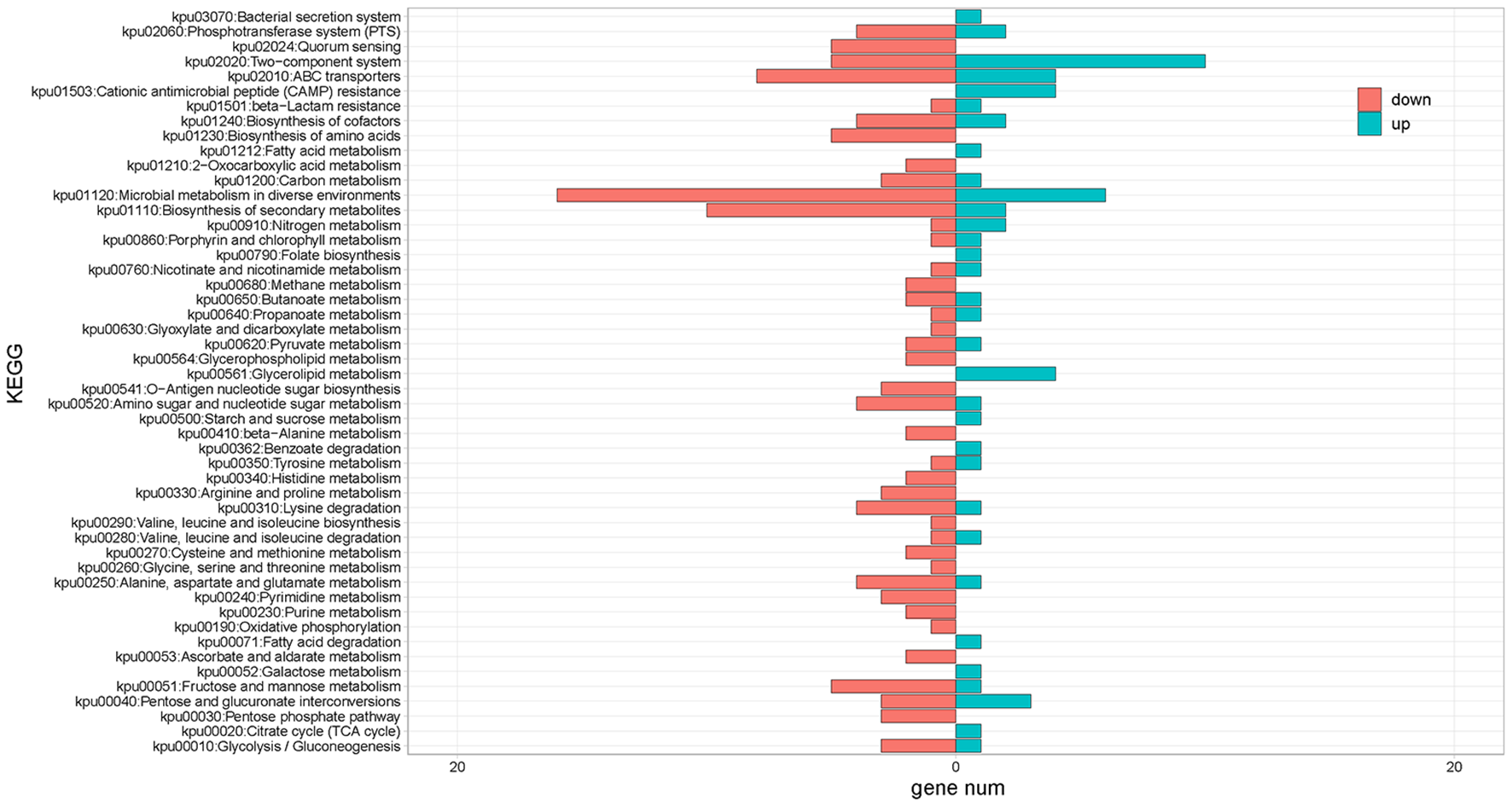

Supplement: Supplementary Figure 4 — KEGG analysis of the DEGs shared by proteome and transcriptome. Red and blue columns represent the number of down-regulated and up-regulated DEGS, respectively. [file Image_4.TIF]
