# Supplementary material for: Proteomic and Transcriptomic Analyses Indicate Reduced Biofilm-Forming Abilities in Cefiderocol-Resistant Klebsiella pneumoniae
Source: Front Microbiol. 2022 Jan 3;12:778190. doi: 10.3389/fmicb.2021.778190 (PMC8762213; doi:10.3389/fmicb.2021.778190)
Supplement: Supplementary file 5 [file Data_Sheet_1.DOCX]

**Supplementary File 1| Transcriptome analysis**

1. **Sample collection and preparation**
   1. **RNA quantification and qualification**

RNA degradation and contamination was monitored on 1% agarose gels. Total amounts and integrity of RNA were assessed using the RNA Nano 6000 Assay Kit of the Bioanalyzer 2100 system (Agilent Technologies, CA, USA). (Refer to RNA quality inspection report for specific instruments).

- 1. **Library preparation for Transcriptome sequencing**

Total RNA was used as input material for the RNA sample preparations. For prokaryotic samples, mRNA was purified from total RNA by using probes to remove rRNA. Fragmentation was carried out using divalent cations under elevated temperature in First Strand Synthesis Reaction Buffer (5×). First strand cDNA was synthesized using random hexamer primer and M-MuLV Reverse Transcriptase, then use RNaseH to degrade the RNA. And in the DNA polymerase I system, use dUTP to replace the dNTP of dTTP as the raw material to synthesize the second strand of cDNA. Remaining overhangs were converted into blunt ends via exonuclease/polymerase activities. After adenylation of 3’ ends of DNA fragments, Adaptor with hairpin loop structure were ligated to prepare for hybridization. Then USER Enzyme was used to degrade the second strand of cDNA containing U, in order to select cDNA fragments of preferentially 370~420 bp in length, the library fragments were purified with AMPure XP system (Beckman Coulter, Beverly, USA). Then PCR amplification, the PCR product was purified by AMPure XP beads, and the library was finally obtained.

In order to ensure the quality of the library, the library needs to be tested. After the construction of the library, the library was initially quantified by Qubit 2.0 Fluorometer, then diluted to 1.5 ng/uL, and the insert size of the library is detected by Agilent 2100 bioanalyzer. After insert size meets the expectation, qRT-PCR is used to accurately quantify the effective concentration of the library (the effective concentration of the library is higher than that of 2 nM) to ensure the quality of the library.

- 1. **Clustering and sequencing**

After the library is qualified, the different libraries are pooling according to the effective concentration and the target amount of data off the machine, then being sequenced by the Illumina NovaSeq 6000. The end reading of 150 bp pairing is generated. The basic principle of sequencing is to synthesize and sequence at the same time (Sequencing by Synthesis). Four fluorescent labeled dNTP, DNA polymerase and splice primers were added to the sequenced flowcell and amplified. When the sequence cluster extends the complementary chain, each dNTP labeled by fluorescence can release the corresponding fluorescence. The sequencer captures the fluorescence signal and converts the optical signal into the sequencing peak by computer software, so as to obtain the sequence information of the fragment to be tested.

**2. Data Analysis**

**2.1 Quality control**

The image data measured by the high-throughput sequencer are converted into sequence data (reads) by CASAVA base recognition. Raw data (raw reads) of fastq format were firstly processed through in-house perl scripts. In this step, clean data (clean reads) were obtained by removing reads containing adapter, reads containing N base and low quality reads from raw data. At the same time, Q20, Q30 and GC content the clean data were calculated. All the downstream analyses were based on the clean data with high quality.

**2.2 Reads mapping to the reference genome**

Reference genome and gene model annotation files were downloaded from genome website directly. Both building index of reference genome and aligning clean reads to reference genome were used Bowtie2 (v.2.3.4.3).

**2.3 Novel gene and gene structure analysis**

Rockhopper (v.1.2.1) software was used to identify novel genes, operon, TSS, TTS and Cis-natural antisense transcripts. It can be used for efficient and accurate analysis of bacterial RNA-seq data, and that it can aid with elucidation of bacterial transcriptomes. Then, we extract upstream 700 bp sequence of Transcription Start Site for predicting promoter using TDNN. (Time-Delay Neural Network)

**2.4 Predict UTR**

According to the information of Transcription Start Site (Transcription terminal Site) and Translation start site (Translation terminal site), we extracted 5’UTR (3’UTR) sequences. Then, RBSfinder (v.1.0) and TransTermH (v.2.0.9) were used to predict SD sequence and terminator sequence respectively.

**2.5 Analysis of sRNA**

Rockhopper was used to identify new intergenic region transcripts, and Blastx was compared with the nr library to annotate the newly predicted transgenic regions, and the unmarked transcripts were used as candidate non-coding sRNAs. RNA fold (v.1.8.5) and Inta RNA (v.1.8.5) were used to predict secondary structure and target gene respectively.

**2.6 Quantification of gene expression level**

HTSeq (v.0.9.1) was used to count the reads numbers mapped to each gene. And then FPKM of each gene was calculated based on the length of the gene and reads count mapped to this gene. FPKM, expected number of Fragments Per Kilobase of transcript sequence per Millions base pairs sequenced, considers the effect of sequencing depth and gene length for the reads count at the same time, and is currently the most commonly used method for estimating gene expression levels.

**2.6 Differential expression analysis**

Differential expression analysis of two conditions/groups (two biological replicates per condition) was performed using the DESeq2 R package (v.1.20.0). DESeq2 provide statistical routines for determining differential expression in digital gene expression data using a model based on the negative binomial distribution. The resulting *P* values were adjusted using the Benjamini and Hochberg’s approach for controlling the false discovery rate. *P* < 0.05 and | log2(foldchange) | > 0 were set as the threshold for significantly differential expression.

**2.7 GO and KEGG enrichment analysis of differentially expressed genes**

Gene Ontology (GO) enrichment analysis of differentially expressed genes was implemented by the cluster Profiler R package (v.3.8.1), in which gene length bias was corrected. GO terms with corrected P value less than 0.05 were considered significantly enriched by differential expressed genes. KEGG is a database resource for understanding high-level functions and utilities of the biological system, such as the cell, the organism and the ecosystem, from molecular-level information, especially large-scale molecular datasets generated by genome sequencing and other high-through put experimental technologies (http://www.genome.jp/kegg/). We used cluster Profiler R package to test the statistical enrichment of differential expression genes in KEGG pathways.
