# Supplementary material for: Proteomic and Transcriptomic Analyses Indicate Reduced Biofilm-Forming Abilities in Cefiderocol-Resistant Klebsiella pneumoniae
Source: Front Microbiol. 2022 Jan 3;12:778190. doi: 10.3389/fmicb.2021.778190 (PMC8762213; doi:10.3389/fmicb.2021.778190)
Supplement: Supplementary file 6 [file Table_1.DOCX]

**Supplementary Table S1** | The changes of OD_590_ in the WT strains and the cefiderocol-treated strains during 18h

| Time | The WT strains | | The cefiderocol-treated strains | | *P* value |
| --- | --- | --- | --- | --- | --- |
|  | average | SD | average | SD |  |
| 1h | 0.052 | 0.002 | 0.058 | 0.003 | 3.16E-12 |
| 2h | 0.061 | 0.004 | 0.072 | 0.014 | 0.000012 |
| 3h | 0.110 | 0.019 | 0.142 | 0.027 | 2.40E-10 |
| 4h | 0.295 | 0.036 | 0.274 | 0.028 | 0.006159 |
| 5h | 0.414 | 0.020 | 0.328 | 0.022 | 0.00E+00 |
| 6h | 0.486 | 0.024 | 0.378 | 0.024 | 0.00E+00 |
| 7h | 0.560 | 0.014 | 0.440 | 0.021 | 0.00E+00 |
| 8h | 0.621 | 0.018 | 0.471 | 0.020 | 0.00E+00 |
| 9h | 0.656 | 0.013 | 0.499 | 0.019 | 0.00E+00 |
| 10h | 0.682 | 0.013 | 0.510 | 0.025 | 0.00E+00 |
| 11h | 0.663 | 0.013 | 0.496 | 0.025 | 0.00E+00 |
| 12h | 0.690 | 0.010 | 0.518 | 0.034 | 0.00E+00 |
| 13h | 0.701 | 0.010 | 0.516 | 0.035 | 0.00E+00 |
| 14h | 0.695 | 0.010 | 0.510 | 0.028 | 0.00E+00 |
| 15h | 0.718 | 0.011 | 0.501 | 0.026 | 0.00E+00 |
| 16h | 0.711 | 0.011 | 0.492 | 0.0372 | 0.00E+00 |
| 17h | 0.716 | 0.008 | 0.48355 | 0.03606 | 0.00E+00 |
| 18h | 0.699 | 0.010 | 0.45425 | 0.03812 | 0.00E+00 |
