# Supplementary material for: Proteomic and Transcriptomic Analyses Indicate Reduced Biofilm-Forming Abilities in Cefiderocol-Resistant Klebsiella pneumoniae
Source: Front Microbiol. 2022 Jan 3;12:778190. doi: 10.3389/fmicb.2021.778190 (PMC8762213; doi:10.3389/fmicb.2021.778190)
Supplement: Supplementary file 7 [file Table_2.DOCX]

**Supplementary Table S2 |** Comparison of total carbon metabolism ability between the WT and cefiderocol-treated strains in 6 time periods (24 h, 48 h, 72 h, 96 h, 120 h and 144 h).

| Time | The WT strains | | | The cefiderocol-treated strains | | | *P* value |
| --- | --- | --- | --- | --- | --- | --- | --- |
|  | average | SD | | average | SD | |  |
| 0h | 0.226 | | 0.005 | 0.123 | | 0.002 | 0.000004 |
| 24h | 3.664 | | 0.014 | 1.162 | | 0.008 | 1.02E-09 |
| 48h | 14.594 | | 0.044 | 10.313 | | 0.033 | 1.80E-08 |
| 72h | 11.562 | | 0.043 | 9.537 | | 0.033 | 3.36E-07 |
| 96h | 4.478 | | 0.023 | 11.220 | | 0.054 | 3.92E-09 |
| 120h | 12.988 | | 0.056 | 13.568 | | 0.060 | 0.000258 |
| 144h | 9.913 | | 0.070 | 19.161 | | 0.082 | 1.26E-08 |
