# Supplementary material for: Proteomic and Transcriptomic Analyses Indicate Reduced Biofilm-Forming Abilities in Cefiderocol-Resistant Klebsiella pneumoniae
Source: Front Microbiol. 2022 Jan 3;12:778190. doi: 10.3389/fmicb.2021.778190 (PMC8762213; doi:10.3389/fmicb.2021.778190)
Supplement: Supplementary file 8 [file Table_3.DOCX]

**Supplementary Table S3 |** Utilization rates of 6 kinds of carbon sources for the WT strains and cefiderocol-treated strains in 6 time periods (24 h, 48 h, 72 h, 96 h, 120 h and 144 h)

| Carbon sources | The WT strains | | | The cefiderocol-treated strains | | *P* value |
| --- | --- | --- | --- | --- | --- | --- |
|  | average | | SD | average | SD |  |
| Phenolic compound | 0.252 | 0.111 | | 0.046 | 0.030 | 0.000477 |
| Amine | 0.499 | 0.213 | | 0.504 | 0.325 | 0.977132 |
| Carboxylic acid | 1.540 | 1.481 | | 2.153 | 1.160 | 0.406018 |
| Carbohydrate | 5.586 | 1.941 | | 6.108 | 2.834 | 0.695161 |
| Amino acid | 1.418 | 0.575 | | 2.086 | 1.224 | 0.215326 |
